# 

**Published:** 1891-04-18

**Authors:** 


### NOTICE TO CORRESPONDENTS.

All MS., Letters, Books for Review, and other matters intended for the Editor should be addressed The Editor, The Lodge, Porchester Square, London, W.

The Editor cannot undertake to return rejected M.S., even when accompanied by stamped directed envelope.

**Notice to Advertisers and Subscribers.**

All Advertisements, Orders for Copies of The Hospital, and Business Communications should be addressed to The Publisher, *not to the Editor*, The Hospital, 140, Strand, W.C.

The Cost of Subscription, post free, is as follows:—Per week, 1½d.; per quarter, 1s. 8d.; per half-year, 3s. 3d.; per annum, 6s. 6d. Foreign:—Per annum, 8s. 8d.; payable, in all cases, in advance.

### General Charities.

[This Column is devoted to an account of work of charitable institutions other than Medical Charities. The Editor will be glad to receive reports or news of work at 140, Strand. *Philanthropy* should be plainly written on the envelope.]

At the 133rd anniversary festival in aid of the funds of the **ORPHAN WORKING SCHOOL AND ALEXANDRA ORPHANAGE,** at Haverstock Hill and Hornsey Rise, which took place last week, the Secretary was able to announce £1,500 in donations, including twenty guineas from the Chairman, H.R.H. the Duke of Cambridge.

The 21st annual ball in aid of the **METROPOLITAN AND CITY POLICE ORPHANAGE** was held last week at the Cannon Street Hotel, under the patronage of the Lord Mayor and Lady Mayoress. This Institution has 256 children in the orphanage, and is giving 2s. 6d. a-week to 669 children in their own homes. Only the children of subscribers to the Institution are benefited. Donations gladly received by the Secretary, Wellesley House, Twickenham, S.W.

The festival of **ST. JOHN'S FOUNDATION SCHOOL FOR SONS OF POOR CLERGY,** Leatherhead, takes place on Wednesday, April 22nd, the Postmaster-General in the chair. This well-known school is appealing for especial help this year in consequence of the outlay which has been made in the extension of the buildings of the school. The small incomes of this majority of the clergy are proverbial, and the difficulties of unpaid tithe and unlet glebe of late years have considerably added to the majority. The school maintains and educates from nine to fifteen years of age the sons of clergy now living. Last year 180 boys were benefitted. Donations will be gladly received by the Secretary, 1, The Sanctuary, Westminster.

The Directors of the **CITY OF LONDON GENERAL PENSION SOCIETY,** 31, Finsbury Circus, E.C., which was founded seventy-three years ago with the object of affording permanent assistance by granting monthly pensions, the individual amounts of which are not stated, to poor mechanics and tradesmen who are over sixty years of age, and who, through decrepitude or illness, have lost their power of self-support, appeal to the public for donations. They claim, we believe correctly, that the Institution, though solely supported by voluntary contributions, has been fortunate enough not to have found it necessary to make such appeals on many previous occasions. The cause of the present appeal is the fact that the amount of subscriptions and donations during the past few years has been altogether inadequate to meet many cases which have been commended to their sympathy. The result is that at the present time a large number in genuine distress are anxiously awaiting *election* in order to share the benefits granted by the institution, which are extended to the widows of pensioners. This system of election means doubled and unnecessary anxiety to many worthy people who are passed over, but we fear that the sadness of their lot does not find its way to the hearts of those to whom the possession of five pounds' worth of brief authority is of far greater value than the bringing of happiness to other people's lives.

Two and a half years ago **THE LONDON FACTORY LADS' CHRISTIAN HOME AND INSTITUTE** was established to meet an educational want amongst children at the age of 13 to 17 years. Sir Richard Temple—and he should be a reliable authority on such matters—holds that our system of elementary education has met with a wonderful measure of success up to the age of 13, and that the country has succeeded in imparting useful and practical instruction to children below that age. Like others who have had a wide experience of the world, he has found that the age of 13 is a critical period in a child's life; it is the moment when the moorings of home influence, which have held them firmly and gently, are suddenly snapped, and the children are cast adrift on a world of debasing and sordid surroundings. The demoralising influence of factory life in all large towns is too well known to need more than a passing notice, and its evils are so great that some have called in the Legislature to interfere. But the power of legislation must ever pale by the side of private agencies in the work of battling against such evils as have called this institution into existence. Every year, according to trustworthy computation, fully 70,000 children leave the schools in the metropolis of London alone, and enter upon the actualities of life without any further guidance, of whom 40,000 are under 13 years of age. Some remedy for the evil has been provided by the organisation on the part of the School Board of evening classes, where useful knowledge and instruction in the industrial arts are imparted. As a supplement to this, the institution provides affectionate care and homeful nursing attendance for many waifs and strays, who not only have been born in London, but have come there from various parts of the country. During the past year the superintendents have endeavoured to shield from the numerous tempations which beset such children as many as their small funds permit. Thirteen fresh lads, according to the latest report, nearly all of them orphans who have been unable to support themselves, have been taken during the past year into the home, and nearly 10,000 attendances of factory lads were recorded. Mr. and Mrs. Markham appeal for donations in aid of the extension of the work of the institute at 1, Patshull Road, Kentish Town, N.W.

### Scraps and Gleanings.

THE epidemic of influenza in Chicago is now subsiding.

A WOMAN of Downpatrick was returned as aged 107 years on the census paper.

A YOUNG man, who fell with his wrist under him whilst playing football in Southport, has died of lockjaw.

A BALL was given at Brighton on the 3rd inst. in aid of the funds of the Hospital for Women and Children.

LEICESTER Provident Dispensaries now have 32,587 members; there is still a balance due to the Treasurer of £1,072.

A NEW illustrated periodical entitled "The Ludgate Monthly," will appear on May 1st. The price is to be only threepence.

DR. YOUNGER, of Hanwell Asylum, has been presented by the nurses with a handsome salver, on the occasion of his resignation.

AN "Association for Preventing the Immigration of Destitute Aliens" has been formed, for dealing generally with this question.

THERE is an average of nearly 5,000 unskilled workmen now in daily employment upon Mr. Balfour's light railways in the West of Ireland.

A VETERINARY surgeon of Parndorf, near Pressburg, has died of blood poisoning, contracted while killing some horses suffering from glanders.

MR. A. T. BRIGHT, M.P., has been appointed a member of the Royal Commission on Vaccination in the place of the late Mr. Charles Bradlaugh.

THE annual report of the Minister of Indian Affairs in Canada, states that there has been a large decrease of Indians in some of the provinces, due to influenza.

WITH reference to a paragraph in this column last week, Mr. A. J. Sewell, of 55, Elizabeth Street, S.W., writes to say that he is not the Mr. Sewell referred to.

IN consequence of the strike of jurymen in South London, Mr. Wyatt, the Coroner, has directed his officers to summon eighteen jurymen on every case instead of the usual twelve.

INFLUENZA—dreaded Russian influenza—is epidemic in Sheffield, and becoming more common in London. Let us hope it will be at least of a milder form than the awful scourge of last year.

IRRATIONAL ladies in rational dress held a bazaar on Tuesday and Wednesday at Kensington. They looked neat and not gaudy, but we refuse to believe it was "the coming dress" they wore.

A BAZAAR in aid of the building fund of the proposed new infirmary at Stafford, was opened on the 2nd inst. The takings of the bazaar amounted to £872 7s. 3d. on the first day, and to £360 on the second day.

THE Duke of Cambridge presided at the thirty-third anniversary festival of the Orphan Working School and Alexandra Orphanage, which was held on the 9th inst. Donations amounting to £1,500 were announced.

AT a meeting convened by the Duke of Devonshire to further the project of a new general infirmary for Derbyshire, it was announced that the Queen would lay the foundation stone of the new buildings on May 21st.

THE financial statement of the Meath Hospital and co. Dublin Infirmary shows that the adverse balance of £187 4s. 3d. with which the year opened has been increased by a deficiency in the accounts for 1890 of £336 14s. 2d.

A FRENCH military doctor has written a book advocating more independent movement of the medical corps when attached to an army in the field, greater mobility of all its parts, and the lightening and lowering of its carriages.

DONATIONS amounting to about £4,700 were announced at the seventy-eighth anniversary festival of the London Orphan Asylum, held at the Hotel Metropole. Of this sum 200 guineas were collected by old boys and girls of the institution.

A "GENERAL Practitioners'" Union is being formed, having, among other objects, "to consider the question of the abuse of medical charities to obtain evidence thereon, and to take such steps as may be necessary to safeguard the interests of general practitioners.

A FRIEND of the Royal Albert Orphan Asylum has promised a donation of £1,000 towards the reduction of the mortgage on the Asylum, on condition that £3,000 more is raised before the festival dinner on the 22nd inst., at which the Duke of Clarence and Avondale will preside.

DR. BERNARD HOFFMEISTER, physician to the Queen, who fell from his horse on March 24th, succumbed to his injuries at Osborne on April 9th. He was only thirty-two years of age, and was a son of the late Sir William Hoffmeister, who was physician to the Queen for many years.

THE late Miss Anna Tucker bequeathed one-half of her residuary estate to the Committee of the Lyme Regis Cottage Hospital, and she directed the other half to be invested in the names of the official trustees of charity funds, the dividends to be applied yearly, at Christmas, in the distribution of bread, coal, or clothing among the poor of Lyme Regis.

IN connection with the extensive works now in progress at Aldershot camp, with the purpose of providing accommodation for an increased number of troops, it has been decided to make important additions to Cambridge Hospital, to meet the greater demands for medical treatment. Major-General Carr-Glyn has been appointed President of the Board charged with the selection of sites for the new hospital buildings.

A FETE is to be held at the Kensington Town Hall, on April 22nd, 23rd, 24th, and 25th, under the patronage of Princess Louise (Marchioness of Lorne), Princess Christian, the Duchess of Teck, and nearly a hundred other ladies. It is most appropriately named, "A Dream of Health for Sick Children," and the proceeds are to go towards furnishing the Convalescent Home at Broadstairs for the poor little invalid children of the Victoria Hospital.

THE metropolis has been visited by two separate outbreaks of small-pox, which has already resulted in two deaths. A dock labourer, who had been employed in unloading foreign grain, was attacked by the disease. A few days after his removal to the hospital ship "Atlas," five persons living in the same house were attacked, and had to be removed also, and a casual acquaintance who had sat by the dock labourer was attacked 13 days afterwards. Another case arose in another part of London. The patient in this case is supposed to have contracted it in the provinces.

### THE STUDENT OF MEDICINE.

[All communications for this Supplement should be addressed to The Editor of The Hospital, at 140, Strand, with the words, "Students' Column," written in the left hand top corner of the envelope.]

### LECTURES: THEIR USE AND ABUSE.—*(Concluded.)*

By H. Wolseley Lewis.

In the second part of this paper I want to say something about the use and abuse of lecturing, apart from the consideration of the subject on which the lecture should be given. There is an old Arabic saying which divides men into four classes, and wh ich has been translated into English thus:—

He who knows not, and knows not that he knows not,
He is a fool—shun him.
He who knows not, and knows that he knows not,
He is simple—teach him.
He who knows, and knows not that he knows,
He is asleep—wake him.
He who knows, and knows that he knows,
He is wise—follow him.

Here it is suggested that you should teach him who knows not, and knows that he knows not, and it appears good advice, because he will almost invariably make a good listener. Of course, the lecturer may complain that he cannot choose his hearers, and too often has to lecture to the fool, who knows not, and yet knows not that he knows not, whom this old saying advises him to shun, and for this evil I confess I see no remedy but to convert him into a member of the second class by convincing him that he knows not, and this, I allow, is often very difficult. Here also it is advised that we follow him who knows and knows that he knows because he is wise; and indeed, as far at any rate as knowledge goes, I think we might take this man as our *beau ideal* of a lecturer. Probably the most obvious abuse of lectures is their excess, and this excess is of two kinds: excess in number and excess in length. First as to excess in number, which I have said does not apply to what I called the "practical" form of lecture. I think, allowing the premises that lectures should treat of those things which cannot readily be learnt elsewhere, they should certainly not exceed one or, at most, two per diem, because they must necessarily be committed to memory at once, or reconstructed from what notes the student is enabled to make during the lecture; and, indeed, I may exclude this latter possibility, as, under these circumstances, the notes are, or should be, practically written manuscripts of the lectures, and it would save time to have the lectures printed and read by the student. Secondly, as to excess in length. Professor Max Müller says that an hour is certainly too long for a lecture which requires us to be wide awake from beginning to end, and that it is generally the last quarter of an hour which does all the mischief, and makes us impatient, dissatisfied, and angry, and, indeed, too often undoes all the good that the previous three-quarters of an hour may have done; therefore it is that he says that no lecture should occupy more than forty-five minutes. Perhaps the majority of lecturers err on the side of being, if I may be allowed the expression, "too wordy," as is well indicated by the verse—

"And still his tongue ran on, the less
Of weight it bore, with greater ease;
And with its everlasting clack,
Set all men's ears upon the rack."

Not but that I recognise the fact there are lectures which are too condensed, indeed, so much so that it is not possible even for the most attentive student to remember even a fair proportion of what has been said; but there is a happy mean, which is obtained by the man who is a thorough master of the art of lecturing, an art, it has been said, "which many attempt and few possess." All lecturers have probably had some experience of the student who, almost directly he enters the lecture-room, begins to show signs of uneasiness, begins to shuffle his feet, to talk to his neighbour, to have a most curious aptitude for dropping his note-book, and, indeed, to make himself generally a nuisance. This may be due to the unsympathetic hardness of the form on which he has to sit, or to the draught which blows down between his collar and his neck, or perhaps to some hereditary predisposition to make himself objectionable, but more often, I thin, it is due to a feeling of opposition and irritability caused by his having had to attend so many and such long lectures previously. As I mentioned at the beginning of this paper, it is very difficult for a lecturer to render his lecture profitable to all his listeners, who may vary so widely in their capabilities. Thus, while a

### The Hospital Advertiser.

### THE STUDENT OF MEDICINE—(*continued*).

particular argument may appear perfectly obvious to one, it is quite inconclusive to another, and the only way in which, so far as I can see, this difficulty can be overcome is by the lecturer encouraging his hearers to question him afterwards on those points on which they are not quite clear, otherwise they may often go away with very dim and confused ideas of what he has been saying. There is a story told of a certain lecturer who, having devoted a whole lecture to proving that Hebrew was not the primitive language of all mankind, and having placed before his audience a complete genealogical tree of the Aryan and Semitic languages, was afterwards thanked by one of them for showing so clearly how all languages, including Sanskrit and English, were derived from Hebrew, the language spoken in Paradise by Adam and Eve. As regards the time of day at which lectures should take place, for those treating of matter which must be at once committed to memory, and which can only be obtained from lectures, the best time is such that, while convenient to both lecturer and hearer, the mind is least preoccupied and most receptive; the difficulty, of course, arises that one person can most readily concentrate his mind on a lecture at one time, another at another, this question, I suppose, ought to be decided by the votes of the majority. I would only suggest that in summer, nine a.m. seems to be an excellent time, though this hour would very probably not be convenient to many in the winter. As a student, I know hardly anything which has a more soporific effect than a lecture delivered in a monotone. One of the most marked advantages which a lecture has over a book is derived from the varying inflexions of the speaker's voice; and to strip it of this advantage is a very great error on the part of the lecturer. In conclusion, I would say then: Eminently the use of lectures is this, that while they are delivered in a strain which appeals to all the hearers, in a tone which carries its meaning with it, at a suitable time, and in a suitable place, while not too numerous and not too long, they should treat of a subject which is not so readily learnt elsewhere; and they should be delivered by a lecturer who is anxious to impart his knowledge to his hearers, who in their turn are anxious to obtain it from him. And when such are the lectures commonly delivered at our medical schools and elsewhere, there will not be so much need to read another paper on "Lectures: their Use and Abuse."

### STUDENTS' MEDICAL SOCIETIES.

#### Middlesex Hospital Medical Society.

The annual general meeting was held on Thursday, March 12th. Mr. A. Pearce Gould, the retiring President, was in the chair. Mr. J. Bland Sutton, F.R.C.S., was unanimously elected President for the next year, Mr. Kenneth Lawson as Secretary, and Mr. H. Bankes-Price as Treasurer. Dr. W. E. Wynter and Mr. G. Brodie were re-elected as vice-presidents. The Treasurer and Secretary then read their reports for the past year. Mr. W. A. Ward read a paper entitled "Hypnotism and its Relation to Medicine," describing the history of the science from its earliest infancy up to the present time, its claims upon our recognition and the good it had already wrought. He thought that the medical profession had not given it a fair trial, and suggested that the Government should appoint a Commission to thoroughly examine and criticise the results hitherto obtained by those who have practised it. The President, Messrs. T. T. Cockill, Sumner, and Best took part in the discussion which followed, and Mr. Leopold Hudson showed to the Society Dr. Luy's "Hypnotic Clock," as used by Professor Charcot, explaining its use, and describing the effects which he had witnessed in his use of hypnotism in private practice. The meeting closed with a vote of thanks to the President, and to Mr. Ward for the paper he had read.

### APPOINTMENTS.

At the Royal Free Hospital, Gray's Inn Road, E. B. Waggett, M.B., B.C., B.A. Camb., M.R.C.S., has been appointed junior resident medical officer. At Charing Cross Hospital, Archie T. Collum, L.R.C.P., M.R.C.S., has been appointed house physician; Charles Gibbs, L.R.C.P., M.R.C.S., house surgeon; and H. G. H. Parham, M.R.C.S., L.R.C.P., surgical registrar. At the London Lock Hospital and Asylum, Harrow Road, Henry E. Juler, F.R.C.S., has been appointed consulting ophthalmic surgeon. At the Great Northern Central Hospital, Leonard Remfry, M.A., M.D., B.C. Camb., M.R.C.P., has been appointed obstetric physician to out-patients. At the St. Pancras and Northern Dispensary, George Edward Younger, M.D. Brux., M.R.C.P. Lond., has been ap-

### The Hospital Advertiser.

### THE STUDENT OF MEDICINE—(*continued*).

pointed an honorary physician. At the West London Hospital, Leonard Arthur Bidwell, F.R.C.S. Eng., L.S.A., has been appointed assistant surgeon. At St. Mary's Hospital, James Ernest Lane, F.R.C.S., has been appointed surgeon. At the General Lying-in Hospital, York Road, S.E., H. Sharland Pope, M.B., B.C. Camb., has been appointed house physician. At the Bradford Infirmary, Adolph Bronner, M.D. Heidelb., M.R.C.S., has been appointed laryngologist; Hermann Bonner, M.D. Strasburg, and Thomas Whilmot, L.R.C.P., M.R.C.S., honorary assistant medical officers; and C. F. M. Althropp, M.B., L.R.C.P., M.R.C.S., assistant surgeon; also John Lacy Firth, M.D. Lond., M.R.C.S., house surgeon, and Hy. Norman Duncan Milligan, M.B., C.M. Edin., dispensary surgeon. At the Windsor Royal Infirmary, Herbert Fraser, L.R.C.P., M.R.C.S., has been appointed house surgeon. At the Sussex County Asylum, Hayward's Heath, Alex. Simpson, M.A., M.B., C.M. Aber., has been appointed junior assistant medical officer. At the Jaffray Suburban Hospital, Birmingham, A. Max Sully, L.R.C.P., M.R.C.S., has been appointed resident medical officer. At the Southern Dispensary, Liverpool, James Young, M.D. Glas., has been appointed assistant surgeon. At the Liverpool Southern Hospital, W. A. Betts, M.B., C.M. Edin., has been appointed junior house surgeon. At the Birmingham General Hospital, Sir B. Walter Foster, F.R.C.P., M.D. Erlang., M.P., has been appointed consulting physician. At the District Infirmary, Ashton-under-Lyne, William J. Hancock, B.Sc., L.R.C.P., M.R.C.S., has been appointed house surgeon. At the Queen's College, Birmingham, William T. Madin, L.D.S., R.C.S., has been appointed dental tutor. At the Belfast Royal Hospital, John Smythe Marrow, M.D. Edin., M.R.C.S., has been appointed house physician. At the Jervis Street Hospital, Dublin, Joseph Dallas Pratt, M.D. Dubl., F.R.C.S.I., has been appointed surgeon. At the County Asylum, Rainhill, near Liverpool, Ernest Wills, M.B. Lond., M.R.C.S., L.R.C.P., has been appointed assistant medical officer.

### VACANCIES.

The following vacancies are announced this week, the amount of salary in each case being given after the name of the institution:

#### Resident Medical Officers.

Birmingham City Asylum, Rubery Hill, near Bromsgrove, Clinical Assistant—Board, &c.

Borough Lunatic Asylum, Portsmouth, Clinical Assistant—Board, &c.

East London Hospital for Children, Shadwell, E., House Physician—Board, &c.

Devonshire Hospital, Buxton, House Surgeon—Salary £100 and Board, &c. Also Assistant House Surgeon—Salary £50 and Board, &c.

Guest Hospital, Dudley, Resident Medical Officer—Salary £100 and Board, &c.

General Infirmary, Leeds, two Resident House Surgeons—Board, &c. Also a Resident Medical Officer at the Ida Hospital—Honorarium £25.

Holloway Sanatorium Hospital for the Insane, Clinical Assistant—Board, &c.

Hospital for Consumption and Diseases of the Chest, Brompton, Resident Medical Officer—Salary £200 and Board, &c.

North-West London Hospital, Kentish Town Road, Resident Medical Officer, and Assistant Resident Medical Officer.

Newcastle-upon-Tyne City Lunatic Asylum, Gosport, Assistant Medical Officer—Salary £100 and Board, &c.

Parish of Birmingham (Workhouse Infirmary), Medical Clinical Clerk—Honorarium 15 guineas and Board, &c.

Grantham Friendly and Trade Societies' Medical Institution, Resident Medical Officer—Salary £150, &c.

Manchester Hospital for Consumption and Diseases of the Throat, Resident Medical Officer at Bowdon, Cheshire—Salary £60 and Board, &c.

Oxford Eye Hospital, House Surgeon—Honorarium £50 and Board, &c.

Paddington Infirmary, 285, Harrow Road, W., Resident Clinical Assistant—Honorarium and Board, &c.

Salford Union, Assistant Medical Officer for the Union Infirmary, Hope, near Eccles—Salary £130, &c.

Sussex County Hospital, Brighton, House Surgeon—Salary £120 and Board, &c.

Swansea Hospital, Resident Medical Officer—Salary £100, and Board &c.,

West Bromwich District Hospital, Assistant House Surgeon—Board, &c.

West Riding Lunatic Asylum, Wakefield, Pathologist and Assistant Medical Officer—Salary £100 and Board, &c.

#### Non-Resident Medical Officers.

Cancer Hospital (Free), Assistant Surgeon.

Chelsea Hospital for Women, Honorary Pathologist.

Charing Cross Hospital, Surgical Registrar—Salary £40.

General Hospital, Birmingham, Honorary Physician.

Norfolk and Norwich Hospital, Norwich, Physician.

Queen's College, Birmingham, Professor of Medicine.

St. George's and St. James' Dispensary, King Street, Regent Street, Honorary Physician.

Chelsea Medical Officer of Health, Food and Drug Analyst, and Gas Examiner—Salary £400.

### The Hospital Advertiser.

### THE STUDENT OF MEDICINE—(continued).

King's College Hospital, London, Sambrooke Medical Registrar. Also a Demonstrator of Public Health.
West London Hospital, Hammersmith Road, W., Assistant Surgeon.

### PASS LISTS.

**Examining Board in England by the Royal Colleges of Physicians and Surgeons.**

The following gentlemen passed the Second Examination of the Board at a meeting of the Examiners on the 4th inst. :—

Physiology only.—Alfred H. Hardcastle, student of Yorkshire College, Leeds ; H. Spencer Hughes, of Queen's College, Birmingham ; J. Alphonsus Dawes, of Queen's College, Manchester ; Stephen L. Martin, of London Hospital and Mr. Cooke's School of Anatomy and Physiology ; H. Myddelton Moore, Horace R. Pring, Henry Knight, Gerald L. Hanwell, Lancelot M. Breton, Malcolm White, and Edgar C. Peru, of St. Thomas's Hospital ; Hamilton Hearnden, John J. Culmer, William J. Burroughs, Alfred Alexander, and C. Melville Greenway, of Guy's Hospital ; Nowriji M. Tarachand, of Bombay and Middlesex Hospital ; A. Eldon Scott, of Middlesex Hospital ; Walter Allingham and John F. H. Iles, of St. George's Hospital ; Robert D. Cox and S. C. Collingwood Fenwick, of St. Mary's Hospital ; John Moses, of London Hospital ; and Herbert Jenkins, of University College.

Passed on the 6th inst. :—

Anatomy and Physiology.—Reginald G. Hann, student of Yorkshire College, Leeds ; Alfred E. Thompson, of Queen's College, Belfast ; Charles Porter, of Queen's College, Cork ; Frederic W. Willway, C. L'Ostle Mall, A. Bloomfield Densham, and A. E. Raif Rutherford, of Bristol Medical School ; Harold Ashton, of Anderson's College, Glasgow ; Joseph Cryer, Samuel A. Archer, W. Hindshaw Budd, and David D. Stewart, of University College, Liverpool ; J. Hedley Marsh, J. Ramsay Bishop, Ernest Knight, and Arthur Walker, of Owens College, Manchester ; Leonard S. Tomkys, of Queen's College, Birmingham : Walter F. Miller and Ernest H. Drake, of Guy's Hospital.

Anatomy Only.—Thomas Astbury and Ambrose H. Palmer, of Queen's College, Birmingham ; John Tilsley W. A. P. A. V. Price, and F. D'A. M. Williams, of Bristol Medical School ; Arthur E. Syme, of Melbourne University ; Flavell Edmunds and Percy Slack, of Sheffield Medical School ; Frank W. Stokes, of University College ; Winthrop T. Talbot, of Boston University and London Hospital.

Physiology Only.—Bruce E. Edge, Richmond Gouldon, Philip Wilkinson, and T. Leiming Webster, of Owens College, Manchester ; and Jas. E. Gordon, of Glasgow University.

Passed on the 7th inst. :—

Anatomy and Physiology.—George V. A. Robertson, Richard L. B. Smith, David Stephenson, and Frederick Walker, students of the Yorkshire, College, Leeds ; F. Ward Crossman, Arthur Hudson, Edward H. Marsh, and Frederick L. Titley, of Bristol School of Medicine ; Kenway T. Williams, Frederick J. V. Hall, T. S. F. Hudson, W. D'Este Emery, J. Copeland Poole, Edward A. Dando, and Gilbert W. Charsley, of Queen's College, Birmingham ; Edwin Quayle, Alan McDougall, Samuel C. Salter, Albert Thorp, William J. Hoyton, and Joseph Law, of Owen's College, Manchester ; Lionel S. Partridge and H. Russell Welsh, of Oxford University ; Clement W. Branson, of Sheffield Medical School ; David Morrison, of University College ; and Marcus S. Paterson, of St. Mary's Hospital.

Anatomy Only.—Charles H. Ackland and Albert T. Morgan, of Bristol Medical School ; William H. Cooper, of Bristol Medical School and Mr. Cooke's School of Anatomy and Physiology ; H. Meredith Harrison, of St. Thomas's Hospital ; and W. Bryce Orme, of University College.

Physiology Only.—Arthur D. Griffiths, of Bristol Medical School ; Stephen Langton, of St. Mary's Hospital.

Passed on the 8th inst. :—

Anatomy and Physiology.—George E. Bircht, G. Hebb Cowan, and Francis W. Walton, students of London Hospital ; Ernest W. Ormerod, William H. Symons, Brice Collyer, and Arthur D. Ducat, of St. Bartholomew's Hospital ; D. Egryan Jones, of Trinity College, Toronto, and St. Bartholomew's Hospital ; Edward T. Jones and A. Wanstall Clarke, of University College ; G. Haylett Lock and Walter H. Hargreaves, of Middlesex Hospital ; A. Winsmore Hooper, Arthur E. Thorpe, and J. Wiseman Laver, of St. Thomas's Hospital ; Reginald A. Cowie, of Cambridge University and St. Thomas's Hospital ; Arthur Bousfield, of King's College ; A. Cecil Hovenden, G. Stanley Hovenden, and Howard W. Graham, of Guy's Hospital ; Arthur G. Bennett, of St. Mary's Hospital and Mr. Cooke's School of Anatomy and Physiology ; Dossabhoy Nowrojee, of Madras Medical College ; and Thomas S. Davies, of St. George's Hospital and Mr. Cooke's School of Anatomy and Physiology.

Anatomy only.—Robert Serjeant, of Guy's Hospital ; Samuel E. Price, of London Hospital ; and Arthur P. Woollright, of St. Bartholomew's Hospital.

Physiology only.—Ernest Shepherd of St. Mary's Hospital ; Louis J. A. de Gebert, of Middlesex Hospital ; Mark R. Taylor, of St. Bartholomew's Hospital ; George W. Gostling, of University College ; John Challice, of London Hospital.

